# Untargetted Metabolomic Exploration of the *Mycobacterium tuberculosis* Stress Response to Cinnamon Essential Oil

**DOI:** 10.3390/biom10030357

**Published:** 2020-02-26

**Authors:** Elwira Sieniawska, Rafał Sawicki, Joanna Golus, Milen I. Georgiev

**Affiliations:** 1 Chair and Department of Pharmacognosy, Medical University of Lublin, Chodzki 1, 20-093 Lublin, Poland; 2 Chair and Department of Biochemistry and Biotechnology, Medical University of Lublin, Chodzki 1, 20-093 Lublin, Poland; rafal.sawicki@umlub.pl (R.S.); joanna.golus@umlub.pl (J.G.); 3 Group of Plant Cell Biotechnology and Metabolomics, The Stephan Angeloff Institute of Microbiology, Bulgarian Academy of Sciences, 139 Ruski Blvd., 4000 Plovdiv, Bulgaria; milengeorgiev@gbg.bg; 4 Center of Plant Systems Biology and Biotechnology, 4000 Plovdiv, Bulgaria

**Keywords:** LC-MS, metabolic pathways, mycobacterial lipids, metabolomics

## Abstract

The antimycobacterial activity of cinnamaldehyde has already been proven for laboratory strains and for clinical isolates. What is more, cinnamaldehyde was shown to threaten the mycobacterial plasma membrane integrity and to activate the stress response system. Following promising applications of metabolomics in drug discovery and development we aimed to explore the mycobacteria response to cinnamaldehyde within cinnamon essential oil treatment by untargeted liquid chromatography–mass spectrometry. The use of predictive metabolite pathway analysis and description of produced lipids enabled the evaluation of the stress symptoms shown by bacteria. This study suggests that bacteria exposed to cinnamaldehyde could reorganize their outer membrane as a physical barrier against stress factors. They probably lowered cell wall permeability and inner membrane fluidity, and possibly redirected carbon flow to store energy in triacylglycerols. Being a reactive compound, cinnamaldehyde may also contribute to disturbances in bacteria redox homeostasis and detoxification mechanisms.

## 1. Introduction

The economic, political, and climate changes in numerous regions of the world are pushing hundreds of thousands of people to flee their homes in the chase for a safer life. This population movement changes the epidemiological condition in countries with a high refugee ratio in terms of promoting the spread of tuberculosis (TB) and other infectious diseases. During the last several years, multidrug-resistant and extremely drug-resistant strains of *Mycobacterium tuberculosis* (Mtb) have been found in every studied country. This fact makes tuberculosis a global threat again [1]. A better understanding of mycobacterial biochemistry is the key to discover new efficient and reliable antituberculosis therapies.

*Mycobacterium tuberculosis* was recently explored by means of metabolomics. Evaluation of sets of metabolites was used to provide a chemotaxonomic characteristic of mycobacteria [2,3]. Metabolomics was also applied for determination of the bacteria’s response to growth arrest [4,5], description of interactions with the host [6], explanation of the mechanism of action of antibiotics and antimycobacterial agents of plant origin [7,8,9], and resistance to standard treatment [10,11,12,13].

Metabolomics is the comprehensive analysis of the set of metabolites, which are the final products of biochemical processes and a result of environmental and genetic interactions in a given biological system. Metabolites can provide information about how the environment affects the organisms that are susceptible to environmental changes and stress conditions [14]. Applications of metabolomics in drug discovery and development has been already shown to be a promising tool to uncover the mechanism of action, efficacy, specificity, or toxicity of lead compounds [15,16,17,18]. Liquid chromatography coupled to mass spectrometry (LC-MS) provides a powerful platform for identification and quantitation of metabolites due to high throughput, soft ionization, and good coverage of small metabolites. In combination with bioinformatics, it enables the understanding of metabolic changes resulting from unusual biological or environmental perturbations [19]. 

Cinnamaldehyde is a natural product found in large quantities in the bark of cinnamon trees (*Cinnamomum verum* J.Presl) and other species of the genus *Cinnamomum*, such as *C. cassia* (L.) J. Presl and *C. camphora* (L.) J. Presl. It can be chemically synthesized; however, the essential oil of cinnamon bark contains up to 90% cinnamaldehyde and its extraction from the essential oil is more economically justified [20]. The antimycobacterial activity of cinnamaldehyde has already been proven in laboratory strains (H37Rv and H37Ra) [21,22] and clinical isolates [23]. What is more, cinnamaldehyde was shown to threaten the mycobacterial plasma membrane integrity and to activate the bacteria stress response system [21]. Because cinnamaldehyde emerged as a potentially active compound, the observation of the bacteria’s response to this stress factor may bring some suggestions how it alters bacterial metabolism. Hence, the aim of this study was to perform an untargeted metabolomic analysis of a set of metabolites produced by *Mycobacterium tuberculosis* H37Ra under the influence of cinnamaldehyde within cinnamon essential oil.

## 2. Materials and Methods 

### 2.1. Mycobacterial Strain and Culture Conditions

#### 2.1.1. Inoculum Preparation

*M. tuberculosis* H37Ra (ATCC25177) was grown on Löwenstein–Jensen slopes (BioMaxima) for up to two weeks. Then the bacterial mass was transferred to 5 mL of the fresh Middlebrook 7H9 broth supplemented with 10% ADC and 0.2% glycerol and was vortexed with 1 mm glass beads for 3 min. After 30 min of sedimentation at room temperature, the upper 2 mL was transferred to a sterile tube and left for the next 15 min. One milliliter of supernatant was placed in a sterile tube and was adjusted to 0.5 McFarland standard with ADC supplemented Middlebrook 7H9 broth [21].

#### 2.1.2. Bacterial Culture Conditions

The 40 mL of Middlebrook 7H9 liquid medium supplemented with ADC enrichment was inoculated with 0.4 ml of prepared inoculum. Bacteria were grown in 50 mL Falcone tubes at 37 °C with 100 rpm, for four to five weeks to obtain the cell density around 1 x 10^9^ CFU/mL (average 50 mg of dry biomass). The test cultures (20 replicates) were supplemented with cinnamon essential oil containing 75% of cinnamaldehyde (CA) to a concentration of 2 mg/mL (normalized by weight, dissolved in DMSO), while DMSO controls (10 replicates) were grown with 2% DMSO and growth control cultures (20 replicates) without any additional treatment. The essential oil composition was determined in our previous study by means of GC-MS [21]. The activity of essential oil against Mtb H37Ra was attributed to the presence of cinnamaldehyde [21]. The effective dose of essential oil for the obtained high-density mycobacterial cultures (approx. 10^9^ CFU/ml) was determined in preliminary experiments (data not shown) and shown to inhibit the growth of bacteria by 50%. The high dose of cinnamaldehyde used in the experiment (2 mg/mL) compared to the minimal inhibitory concentration (MIC) value (8 µg/mL) results from the necessity of employing *M. tuberculosis* cultures with a high cell density [24]. In this study, the cell culture density was 2000 times higher than the density of 5 x 10^5^ CFU/ml used in standard MIC tests. The cultures were incubated for 24 h. Then bacterial metabolism was stopped and metabolites were quenched by addition of cold methanol (−60 °C) (1:1 v/v). Next, the cultures were centrifuged for 30 min at 8000 rpm in 4 °C, and the supernatant was removed. The bacterial pellet was rinsed three times with cold PBS buffer (Biomed, Lublin, Poland) and centrifuged again to remove traces of medium. The bacterial biomass was lyophilized, weighed and stored at −60 °C before analysis.

### 2.2. Liquid Chromatography–Mass Spectrometry (HPLC–ESI–QTOF–MS) Analysis

#### 2.2.1. Metabolite Extraction

Lyophilized samples were extracted to analyze hydrophilic and lipophilic compounds. The mixtures of chloroform and water (1:1 v/v) and methanol and water (1:1 v/v) were used for extraction of lipids and hydrophilic compounds, respectively. The dry biomass (each sample) was poured with a mixture of solvents (1500 µL) and sonicated for 20 min. The samples were centrifuged for 15 min at 13,000 rpm at 4 °C, and 1000 µL of clear solvent mixture was transferred to a clean Eppendorf tube and evaporated under reduced pressure at 30 °C. Dry residue was dissolved in the mobile phase for metabolite analysis.

#### 2.2.2. Chromatographic Conditions

The chromatographic separation was performed on an Agilent 1200 Infinity HPLC chromatograph (Agilent Technologies, Santa Clara, CA, USA) using a thermostated (20 °C) Gemini® chromatographic column (3 µm i.d. C18 with TMS endcapping, 110 Å, 150 x 2 mm) and guard column (Phenomenex Inc, Torrance, CA, USA). The flow rate was set at 0.3 mL/min for the mobile phase A (water containing 0.1% formic acid (v/v)) and mobile phase B (0.1% formic acid in acetonitrile(v/v)). Both mobile phases were mixed in the following gradient program: 5 min, 0% B; 20 min, 66% B; 35 min, 95% B. The stop time was set at 35 min.

#### 2.2.3. Mass Spectrometry Conditions

The MS data were acquired on an Agilent 6530B QTOF Accurate-Mass QTOF spectrometer equipped with Dual Agilent Jet Stream spray source (ESI) (Agilent Technologies, Santa Clara, CA, USA) connected to a N2 generator (Parker Hannifin Corporation, Haverhill, MA; generating N2 at purities > 99%). The following conditions were kept constant in all experiments: drying gas temp: 350 °C, drying gas flow: 12 L/min, sheath gas temp: 400 °C, sheath gas flow: 12 L/min; nebulizer pressure: 40 psig, capillary V (+): 4000 V, skimmer 65 V. The acquisition rate for the MS and MS/MS mode was 2 spectra/s in a scan range from 100 to 1700 m/z. Two spectra were recorded for every feature in the collision energy of 10 and 40 eV. Samples were analyzed in positive (POS) and negative (NEG) modes. For accurate on-line mass calibration, the standard masses (121.0508 and 922.0097 in positive mode; 112.9855 and 966.0007 in negative mode) were injected directly into the ion source.

#### 2.2.4. Data Processing

The acquired data were processed using Mass Hunter Qualitative Analysis (version B.07.00; Agilent Technologies, Santa Clara, CA, USA). Raw data files were converted to mzDATA and feature detection and discovery was performed in open-source software XCMS (version Version 3.7.1; The Scripps Research Institute, La Jolla, CA, USA) applying the centWave algorithm. Further data processing including normalization, scaling and filtering were performed before statistical analysis. Mass traces were only retained if they contained at least three peaks with intensity 500. For retention time correction, the OBI-Warp method was applied. We set 5 ppm as the allowed error and 0.015 m/z as the absolute allowed error for feature annotations. A mass accuracy tolerance of 10 ppm was set as the mass window for the database search. For statistical calculations, the Welch t-test was performed, which is used to compare the means of two independent sample groups with the assumption that two-group variances may differ. The fold change threshold was set as 1.5 and the *p*-value as 0.01 for significant features. Activity network (connections) analysis was performed within XCMS software, applying mummichog version: 1.1.6, (metabolic model *M. tuberculosis* H37Ra, MTUB419947, BioCyc 19.5) with 5 ppm pathway deviation.

Data acquired in positive ESI mode and processed by software XCMS were further analyzed in MS-LAMP software with the integrated “Mtb LipidDB” database for mycobacterial lipids characterization. The m/z values of detected features were assigned to singly protonated ions, (M+H)^+^. A mass window range of 0.05 m/z was allowed for every search. The outputs from MS-LAMP and XCMS were correlated and the number of different up-regulated or down-regulated (compared to untreated group) m/z values assigned to a given lipid class were counted. 

## 3. Results

### 3.1. Changes in Metabolic Profiles

**Figure 1:**
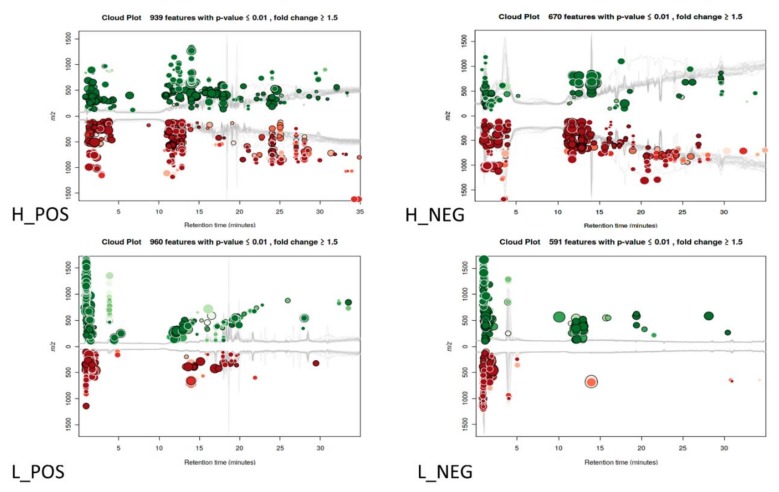
Cloud plot analysis of hydrophilic (H) and lipophilic (L) Mtb whole cell extracts analyzed in positive (POS) and negative (NEG) mode. Fold change > 1.5; *p* < 0.01. The color of the bubble denotes directionality of fold change and the size of the bubble denotes the extent of the fold change. Green color represents up-regulated metabolite features, while red color represents down-regulated metabolite features.

**Figure 2:**
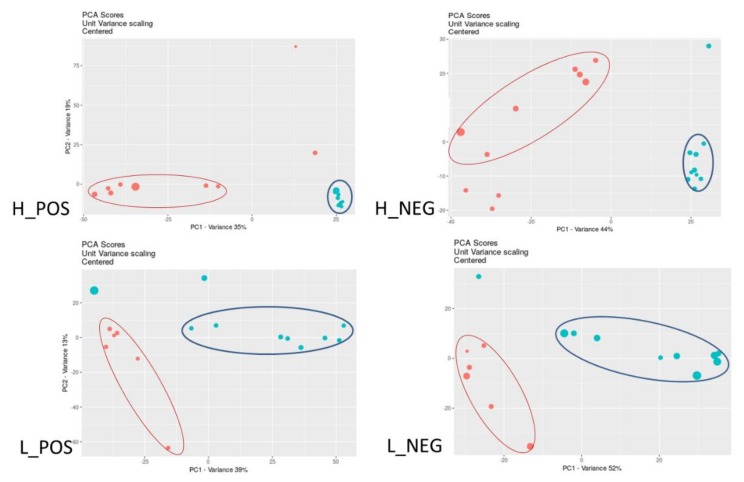
Principal component analysis (PCA) plot of the features detected in test (cinnamaldehyde) and control (untreated) cultures of Mtb; hydrophilic (H) and lipophilic (L) extract; positive (POS) and negative (NEG) mode. Red dots–untreated samples, blue dots–cinnamon essential oil/cinnamaldehyde samples, circles indicate grouped point sets.

The whole cell extracts from Mtb after 24 h exposure were analyzed to identify the changes in metabolite profiles occurring between bacteria exposed to essential oil/cinnamaldehyde and control cultures. Features were defined as a three-dimensional value of m/z, retention time (RT) and intensity (cloud plots). Then, features with equivalent mass and retention time in test and control samples were aligned, which enabled pair-wise comparison of the MS signal intensity to monitor the metabolites that were either increased/decreased or present/absent. Hydrophilic extracts yielded 3886 features (2601 in POS and 1285 in NEG), among which 1609 (939 in POS and 670 in NEG) presented significant variation. Lipophilic extracts yielded 4598 features (3287 in POS and 1311 in NEG), among which 1551 (960 in POS and 591 in NEG) were significantly up- or down-regulated. As can be seen on cloud plots (Figure 1) numerous detected significant features were up- and down-regulated under the influence of essential oil/cinnamaldehyde. Principal component analysis (PCA) (Figure 2) revealed differences between sets of metabolites detected in control and test samples; however, test samples were more consistent in the case of hydrophilic extracts.

Hydrophilic extracts were used for predictive metabolite pathways analysis matching a model of Mtb H37Ra (MTUB419947) in a Kyoto Encyclopedia of Genes and Genomes (KEGG) metabolite repository reference list and showed dysregulation in the production of a number of compounds after essential oil/cinnamaldehyde treatment (Table S1). Significant alterations were obtained for tetrahydrofolate biosynthesis, tRNA charging, factor 420 biosynthesis and biotin biosynthesis from 8-amino-7-oxononanoate I (Table 1).

### 3.2. Dysregulation of Lipids under Essential Oil/Cinnamaldehyde Treatment 

**Figure 3:**
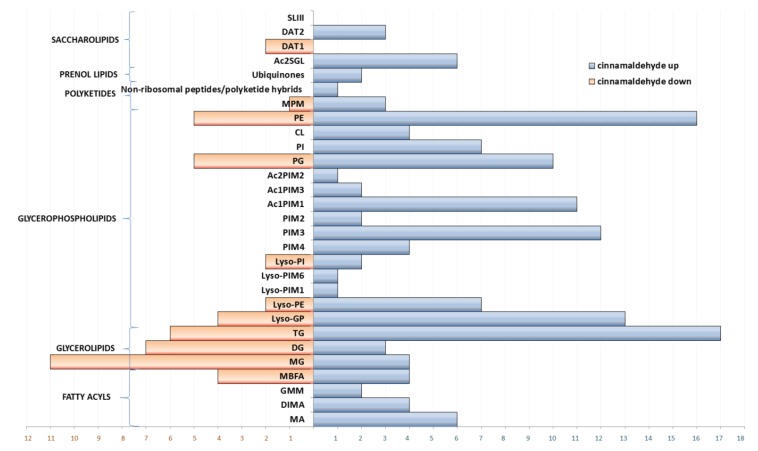
The number of different m/z up-regulated or down-regulated under the influence of CA (versus untreated) assigned to a given lipid class. Blue and red bars represent up-regulated and down-regulated features, respectively. MA–Mycolic acids; DIMA–Phthiocerol dimycocerosates; GMM–Glucose monomycolates; MBFA–Methyl branched fatty acids; MG–Monoacylglycerols; DG–Diacylglycerols; TG–Triacylglycerols; Lyso-GP–Monoacylglycerophosphoglycerols; Lyso-PE–Monoacylglycerolphosphoethanolamines; Lyso-PIM1–Monoacylglycerophosphoinositolmonomannosides; Lyso-PI–Monoacylglycerophosphoinositols; PIM4–Diacylglycerophosphoinositoltetramannosides; PIM3–Diacylglycerophosphoinositoltrimannosides; PIM2–Diacylglycerophosphoinositoldimannosides; Ac1PIM1–Monoacylated diacylglycerophosphoinositolmonomannosides; Ac1PIM3–Monoacylated diacylglycerophosphoinositolmonotrimannosides; Ac2PIM2–Diacylated diacylglycerophosphoinositoldimannosides; PG–Diacylglycerophosphoglycerols; PI–Diacylglycerophosphoinositols; CL–Diacylglycerophosphoglycerophosphodiradylglycerols; PE–Diacylglycerolphosphoethanolamines; MPM–Mannosyl-b1-phosphomycoketides; Ac2SGL–Diacylated Sulfolipid; DAT2–2,3-di-O-acyltrehaloses; DAT1–2,3-di-O-acyltrehaloses; SL-III–Sulfolipid III.

Lipophilic extracts subjected to lipid analysis showed that the majority of detected compounds belonged to nonpolar glycerolipids (24%) and polar glycerophospholipids (57%) (Supplementary file), the latter being major structural components of the mycobacterial plasma membrane and precursors for the outer envelope [25]. Less abundant classes of compounds were fatty acyls, saccharolipids, polyketides and prenol lipids (Supplementary file). The comparison of test samples to control showed predominant upregulation in almost all classes of described lipids (Figure 3). However, downregulation was also observed for glycerolipids, when the number of compounds down-regulated was higher than those up-regulated (monoacylglycerols, MG and diacylglycerols, DG) (Figure 3, Table S2).

#### 3.2.1. Fatty Acyls

In the class of fatty acyls, upregulation was noticed for mycolic acids (MA), phthiocerol dimycocerosates (DIMA) and glucose monomycolates (GMM). The highest number of positively altered lipids belonged to MA, with alpha-MA (C91) being almost twenty times higher (Table S3). These very long-chain fatty acids are found as either esters of an arabinogalactan or as free lipids in the form of trehalose dimycolate (TDM) and contribute to the formation of the mycobacterial outer membrane. Alpha-MA are the most abundant (>70%) within mycolic acids [26]. DIMA and GMM are part of the mycobacterial pseudo-outer membrane and play a role in cell wall permeability [27]. GMM is usually produced in vivo from the host glucose; however, bacteria can acquire it also from glucose rich medium [28]. Beside the structural function, DIMA and GMM play a role in mycobacteria invasion in the host organism [27,28].

#### 3.2.2. Glycerolipids

Glycerolipids, being main apolar lipids and a carbon source in *M. tuberculosis*, were reshuffled from MG and DG to triacylglycerols (TGs). A number of lipids that were up-regulated in the TG group were the highest, and similarly, the highest down-regulation was observed for MG and DG (Figure 3, Table S2). The highest fold change (42 × higher) was noted for glycerol tritetracosanoate (TG 72:0) (Table S3). This redirection of glycerolipid metabolism is correlated with the bacterial response to stress in vitro and in vivo [29,30,31]. TGs are stored in a form of intracellular lipophilic inclusions (ILIs) and are directly linked to division arrest and to increased drug tolerance under stress conditions [32]. TAG synthase encoded by the tgs1(TGS1) gene, which accounts for most of the TAG synthetic activity, was found to be strongly up-regulated in growth-arrested bacilli [31], confirming the possible redirection of carbon flow.

#### 3.2.3. Glycerophospholipids

Upregulation dominated among all detected glycerophospholipids (GPs); however, in five groups of compounds, a small number of molecules were down-regulated (Figure 3; Table S2). Among 15 subclasses, monoacylglycerophosphoglycerol (Lyso-GP 20:0), monoacylated diacylglycerophosphoinositolmonomannoside (Ac1PIM1 46:2), monoacylated diacylglycerophosphoinositoltrimannoside (Ac1PIM3 55:4) and diacylglycerolphosphoethanolamine (PE 34:2) were individual compounds with the highest positive fold change. Glycerophospholipids are the largest class of mycobacterial amphipathic polar lipids, being major structural components of mycobacterial plasma membrane and precursors for the mycobacterial outer envelope [25]. GPs and consequently Lyso-GPs are utilized in bacterial membranes as precursors for cardiolipin (Cl) synthesis [33]. Two Lyso-GP units compose CL, which has four acyl chains, three glycerols and two phosphates and is one of the abundant phospholipids in mycobacteria [28]. Here we detected that Lyso-GP 20:0 was up-regulated almost 14 times (Table S3), whereas cardiolipins were up-regulated up to 7 times (Supplementary file). In the class of phosphoethanolamines (PE) and corresponding mannosides (Lyso-PE), up-regulation was noticed for 16 and 7 compounds, respectively. Some compounds were however down-regulated (Table S2). Diacylglycerolphosphoethanolamine (PE 34:2), playing a structural function in the mycobacterial inner membrane, was up-regulated 44 times compared to control cultures (Table S3). For 10 classes of detected phosphoinositol (PI) related compounds, upregulation was observed for 43 matches, whereas only two compounds were down-regulated (Table S2). PI is a building block for bulk phospholipids of the mycobacterial inner membrane and anchors for linear and mature branched lipomannan (LM) and lipoarabinomannan (LAM). The formation of PI, PIMs and derived products (LM and LAM) serve essential functions in the mycobacterial membrane and cell wall affecting bacterial viability [34]. The highest fold change up, resulting from the influence of cinnamaldehyde, was noted for monoacylatedglycosides: Ac1PIM1 (46:2) (13 × up) and Ac1PIM3 (55:4) (11 × up) (Table S3), being constituents of the inner membrane, with the major lipid (Ac2PIM2) [35] up-regulated six times (Supplementary file).

#### 3.2.4. Polyketides and Prenol Lipids

Slight up-regulation (up to 3 ×) was also detected for polyketides (MPM (C30), MPM (C31), MPM (C34)), which are glycolipids occurring in small amounts in the cell wall of slow-growing mycobacteria only and are related to transmission of signals regulating the rate of cell division, survival, or virulence [36].

Prenol lipids (menaquinones, MK), involved in the mycobacterial respiration and oxidative phosphorylation [37], were up-regulated. An almost 9-fold increase was observed for MK-9, while the sulfated form of MK-9, S881, increased 2.6 times. The transport of electrons and protons from the cytoplasmic side of the membrane to the periplasmic side is the role of MK. Sulfation of menaquinone may be a negative regulator of this process [38].

#### 3.2.5. Saccharolipids

In the class of saccharolipids, all detected acyl forms of sulfolipids (Ac2SGL) were up-regulated, whereas diacyltrehaloses (DAT) were only partially up-regulated (Table S2). Assigned compounds are non-mycoloylated trehalose esters, which mainly contain methyl-branched fatty acids and are present in the outer membrane [39]. The production of polyketide-derived acyltrehaloses is however strongly impaired in the H37Ra strain, because of the mutation in the two-component regulator PhoP-PhoR. Some minimal amounts of diacyltrehaloses esterified with hydroxylated long-chain methyl-branched fatty acids or short-chain unsaturated mono-methyl-branched fatty acids and sulphate group are however preserved [40]. In agreement with this, we detected diacylated sulfolipids (Ac2SGL C57-C74), among which Ac2SGL (C60) was up-regulated 23 times in the test samples (Table S3), while the other forms increased from 1.5 to 8.5 times. Several diacyltrehaloses were also detected. DAT2 was up-regulated up to 8.5 times, whereas DAT1 was only slightly down-regulated (Supplementary file). DAT1 consists of methyl branched and linear chain fatty acids in a ratio of 1:1, whereas DAT2 is composed of linear straight-chain fatty acids and methyl branched and hydroxylated fatty acids in a ratio of 1:1 [41]. Incorporation of methyl branched and hydroxylated fatty acids in DAT was favoured in the test samples.

### 3.3. Changes upon the Influence of DMSO

The main difference in profiles of lipids detected in test samples and samples influenced by DMSO was higher down-regulation in a class of glycerophospholipids. It was manifested by a higher number of compounds present in lower amounts and a high negative fold change observed in Lyso-GP, Lyso-PE and PG for example (Table S3). The highest fold decrease (72 ×) was described for diacylglycerophosphoglycerol (PG 29:1) (C35H67O10P1), containing one short-chain saturated fatty acid and another one with a single double bond. The other observation noticed under the influence of DMSO was the different profile of glycerolipids. MG dominated in this class, and although some significant downregulation was noticed (Table S3), the number of MG that was up-regulated was noticeably higher in comparison to TG.

### 3.4. Essential Oil/Cinnamaldehyde vs. DMSO

To confirm that changes observed under essential oil/cinnamaldehyde treatment are not influenced by DMSO used as a vehicle, these two datasets were analysed together. The profile of lipids in the fatty acyls group obtained for cinnamaldehyde vs. untreated control and cinnamaldehyde vs. DMSO vehicle was similar. The highest up-regulation was observed for MA, then DIMA and GMM. Slight down-regulation was noticed for methyl branched fatty acids, which was not observed for the DMSO influenced group (Figure S2). Likewise, CA vs. DMSO presented the same shift from monoacylglycerols to triacylglycerols as CA vs. untreated (Figure S2). In the glycerophospholipids group, CA vs. DMSO resulted in up-regulation of Lyso-GP, PIM3, Ac1PIM1, CL and PE (PE (37:2) 84 times up). These changes correspond to the positive fold change noticed in CA vs. untreated for Lyso-GP, Ac1PIM1, CL phosphoethanolamines and corresponding mannosides (Supplementary file). The profile of saccharolipids of CA vs. DMSO was also similar to that obtained for CA vs. untreated.

## 4. Discussion

Metabolomic analysis enables the exploration of global metabolic profiles produced by bacteria under stress conditions. Using the untargeted approach, we wanted to evaluate the influence of essential oil/cinnamaldehyde on the set of metabolites produced by *M. tuberculosis* H37Ra. In our previous study we determined that antimycobacterial activity of cinnamon essential oil against Mtb H37Ra is equal to activity of cinnamaldehyde itself [21], hence most probably this compound is primarily responsible for activity of the whole essential oil. DMSO was selected as a second control, as it is known for its action influencing the integrity of bacterial membranes [42]. It was reported to open pores or holes in bacterial cell membranes and increase membrane permeability, facilitating uptake of antibiotics or nucleic acids isolated from bacteriophages [43]. DMSO induces membrane thinning and increases the fluidity of the membrane’s hydrophobic core. It causes desorption of individual lipid molecules from the membrane and consequent disintegration of the bilayer structure [44]. Our results suggest alterations in the composition of mycobacterial membrane lipids (glycerophospholipids) manifested by a reduction in the number of compounds in particular subclasses and a high negative fold change (Figure S1). The noticed changes in lipid profiles may result from direct physico-chemical interactions between DMSO and lipid bilayers. DMSO competes with the lipid head group for favorable interactions with water, and thereby decreases the head group hydrated volume [45] and consequently causes desorption of lipids from the bilayer. However, the dysregulation in the glycerolipid class suggests that additional metabolic changes may also be a reason for altered lipid profiles. The 24 h incubation with influencing substance is sufficient for redirection of metabolism in mycobacterial cells. The observation of an increased number of monoglycerols (MG), which did not however dominate over TG (Figure S1), may suggest changes in GP metabolism leading to production of ILIs, being present in stress conditions in bacteria cells [29,30,31]. Nevertheless, these changes may be delayed in comparison to cultures influenced by essential oil/cinnamaldehyde in which the number of TGs strongly increased after only 24 h of incubation.

Essential oil/cinnamaldehyde caused significant alterations in lipid profiles and metabolic pathways. Pathways analysis, applied in this study, is a tool used to predict dysregulated metabolic routes directly from the accurate mass (m/z) values of the features generated from the processed results. Accurate m/z values and matching adducts are the basis for compound identification by searching against metabolites with predefined adducts in the BioCyc pathway database. This enables one to deconvolve large amounts of metabolic features to a resulting significant list with a significance *p*-value [46]. The highest significant dysregulation after essential oil/cinnamaldehyde treatment was observed for tetrahydrofolate biosynthesis pathway resulting in upregulation of 7,8-dihydropteroate (DHP) and 7,8-dihydrofolate (DHF) (Table 1). Both compounds are involved in tetraydrofolate (THF) synthesis de novo in Mycobacteria. DHF is produced from 7,8-dihydropterin pyrophosphate and para-aminobenzoic acid by DHF synthase, and then reduced by DHF reductase to yield THF [47]. THF enters the folate cycle and is converted to polyglutamylated folates (THF-Glun), which are essential cofactors in the transfer of one-carbon groups in pathways for the synthesis of methionine, N-formylmethionyl-tRNA, glycine, serine, pantothenate, purines, and thymidine [47]. Methionine produced with the participation of THF-Glun is used in the activated methyl cycle to supply the pool of reactive methyl groups involved in the formation of a variety of functional groups in the meromycolic chain of mycolic acids [48]. The number of up-regulated fatty acyls with a fold change up between 1 and 19 observed under essential oil/cinnamaldehyde treatment can be correlated with the pool of reactive methyl groups available for incorporation into mycolic acids. Up-regulation in the tetrahydrofolate biosynthesis pathway may result in increased production of alpha and keto mycolic acids and consequently glucose monomycolates and phthiocerol dimycocerosates. MA, DIMA and GMM are part of the mycobacterial outer membrane and play a role in cell wall permeability [27]. In contrast to MA being covalently bound to arabinogalactan, DIMA are noncovalently attached to the capsule but also contribute to cell envelope architecture and permeability [49]. Bacteria exposed to essential oil/cinnamaldehyde possibly reorganized their outer membrane as a physical barrier against stress. This process may be also supported by up-regulation of phosphoinositol (PI) and related compounds (PIMs) contributing to formation of linear and mature branched LM and LAM. LM and LAM are supposed to fill in pores and cavities present in the peptidoglycan structure, and in this way strengthen the cell wall barrier [50]. The structural defects in LM and LAM result in increased cell wall permeability and increased sensitivity to β-lactam antibiotics, indicating their role in the integrity of mycobacterial cell walls [50]. Essential oil/cinnamaldehyde may possibly induce sealing of the cell wall, but also may cause changes in the composition of the lipid bilayer. The up-regulated production of Ac1PIM1, Ac1PIM3, and especially Ac1PIM2, which predominates in the inner membrane [35], may be related to the regulation of membrane fluidity. The innate mycobacterial inner membrane is characterized by unusually low fluidity because of the presence of densely packed fatty acyl chains within a single molecule [35]. An increased number of these molecules may be needed to restore low membrane fluidity contributing to the general resistance to drugs. By lowering the cell wall permeability and inner membrane fluidity, bacteria may possibly responded to stress caused by essential oil/cinnamaldehyde. Our results are in agreement with the previous observations that essential oil/cinnamaldehyde does not facilitate the action of intracellular antibiotics like streptomycin or rifampicin [21].

Essential oil/cinnamaldehyde possibly down-regulated biotin biosynthesis from 8-amino-7-oxononanoate (Table 1), decreasing the amount of S-adenosyl-4-methylthio-2-oxobutanoate and 8-amino-7-oxononanoate. Dysregulation in this pathway should affect the functions of biotin-dependent enzymes involved in the central carbon metabolism, leading to non-efficient production of fatty acids [51]. However, the observations of lipid profiles of bacteria under essential oil/cinnamaldehyde treatment did not clearly match this assumption. The number of significantly down-regulated molecules was detected in the glycerolipids class (mainly MG), but at the same time, fatty acyls in the subclass of mycolic acids and related compounds were up-regulated. It is quite sound that only partial down-regulation of biotin synthesis will not lead to signification impairment of production of biotin-dependent molecules. Only the Mtb ΔbioA mutant strain, in which the biotin biosynthetic enzyme 7,8-diaminononanoic acid synthase (BioA) was deactivated, was not able to grow without exogenous biotin [52]. Bacteria influenced by essential oil/cinnamaldehyde manifested noticeable fluctuations in the composition of glycerolipids (Figure 3). Down-regulation of MG and DG was contrasted with the high increase in the production of, e.g., glycerol tritetracosanoate, which is stored in a form of intracellular lipophilic inclusions and is an energy deposit under stress conditions. This may suggest the possible redirection of carbon flow, which is directly linked to division arrest and to increased xenobiotic tolerance under stress conditions [32].

The increased xenobiotics tolerance is possibly due to direct detoxification mechanisms represented by the unique bacteria redox cofactor F420 (7,8-didemethyl-8-hydroxy-5-deazariboflavin-5-phosphoryllactyl(glutamyl)n glutamate). Factor F420 is structurally similar to flavin adenine dinucleotide (FAD) and flavin mononucleotide (FMN); however, being an obligate two-electron carrier, it is electrochemically more like nicotinamides NAD and NADP. F420 mediates hydride transfer to or from a wide range of organic carbon compounds [53]. Essential oil/cinnamaldehyde treatment possibly altered the factor 420 biosynthesis pathway, down-regulating 5-amino-6-(d-ribitylamino)uracil, which is essential for synthesis of cofactor F420. However, 5,10-methylene-tetrahydromethanopterin, being a co-product in the production of the reduced form of cofactor F420, was up-regulated, suggesting that perhaps the activated detoxification mechanisms are used under stress conditions. Essential oil/cinnamaldehyde, being a very reactive compound, was proven to increase the activity of superoxide dismutase and glutathione peroxidase in mice [54]. Both enzymes are involved in neutralization of reactive oxygen species (like hydrogen peroxide and the superoxide (O^2−^) radical), and their activity can be compared to F420-dependent NADP oxidoreductases in mycobacteria. Reduction of essential oil/cinnamaldehyde via the action of F420H_2_ may be a simple way to decrease its reactivity. What is more, cofactor F420 was also shown to increase the capacity of mycobacteria to resist antimicrobial agents through mechanisms independent of detoxification [53], which indicates more complicated mechanisms involved in xenobiotic tolerance.

The other observations related to changes in bacteria metabolites under essential oil/cinnamaldehyde treatment were described for polyketides and saccharolipids, which are present in bacteria in very small amounts and have signaling functions. Their importance was not correlated to any action of essential oil/cinnamaldehyde in in vitro culture, because polyketides, as well as saccharolipids, play a key role in virulent bacteria during host infection [36,39].

## 5. Conclusions

Summing up, the current study may suggest that bacteria exposed to essential oil/cinnamaldehyde probably reorganized their outer membrane as a physical barrier against stress. They possibly lowered their cell wall permeability and inner membrane fluidity and may have redirected carbon flow to store energy in triacylglycerols. As cinnamaldehyde is a very active compound and may contribute to disturbances in redox homeostasis, it may also alter detoxification mechanisms.

## Figures and Tables

**Table 1:** Activity network (connections) obtained for bacteria under the treatment of cinnamaldehyde.

KEGG Pathway Name and Metabolites	Total	Hits (Fold Change)	*p*-Value <0.05
tetrahydrofolate biosynthesis:	2	2	0.012
7,8-dihydrofolate		(42.4 up)	
7,8-dihydropteroate		(9.7 up)	
tRNA charging:	4	2	0.03
L-histidine		(3.2 down)	
L-tyrosine		(3.2 down)	
factor 420 biosynthesis:	2	2	0.03863
5-amino-6-(D-ribitylamino)uracil		(9.1 down)	
5,10-methylene-tetrahydromethanopterin		(162.3 up )	
biotin biosynthesis from 8-amino-7-oxononanoate I:	2	2	0.03863
S-adenosyl-4-methylthio-2-oxobutanoate		(4.2 down)	
8-amino-7-oxononanoate		(12.8 down)	

## Supplementary Materials

Figures S1 and S2; Tables S1–S3; Supplementary file, are available online at https://www.mdpi.com/2218-273X/10/3/357/s1.
