# Supplementary material for: Untargetted Metabolomic Exploration of the Mycobacterium tuberculosis Stress Response to Cinnamon Essential Oil
Source: Biomolecules. 2020 Feb 26;10(3):357. doi: 10.3390/biom10030357 (PMC7175327; doi:10.3390/biom10030357)
Supplement: Supplementary file 1 [file biomolecules-10-00357-s001.zip › biomolecules-701621-Supplementary XML Conversion.docx]

Untargetted Metabolomic Exploration of *Mycobacterium tuberculosis* Stress Response to Cinnamon Essential Oil.

Elwira Sieniawska ^1,^*, Joanna Golus ^2^, Rafał Sawicki ^2^ and Milen I. Georgiev^3,4^

^1^ Chair and Department of Pharmacognosy, Medical University of Lublin, Chodzki 1, 20-093 Lublin, Poland

^2^ Chair and Department of Biochemistry and Biotechnology, Medical University of Lublin, Chodzki 1, 20-093 Lublin, Poland; rafal.sawicki@umlub.pl (R.S.); joanna.golus@umlub.pl (J.G.)

^3^ Group of Plant Cell Biotechnology and Metabolomics, The Stephan Angeloff Institute of Microbiology, Bulgarian Academy of Sciences, 139 Ruski Blvd., 4000, Plovdiv, Bulgaria; milengeorgiev@gbg.bg (M.G.)

^4^ Center of Plant Systems Biology and Biotechnology, Plovdiv, Bulgaria

***** Correspondence: esieniawska@pharmacognosy.org (E.S.)


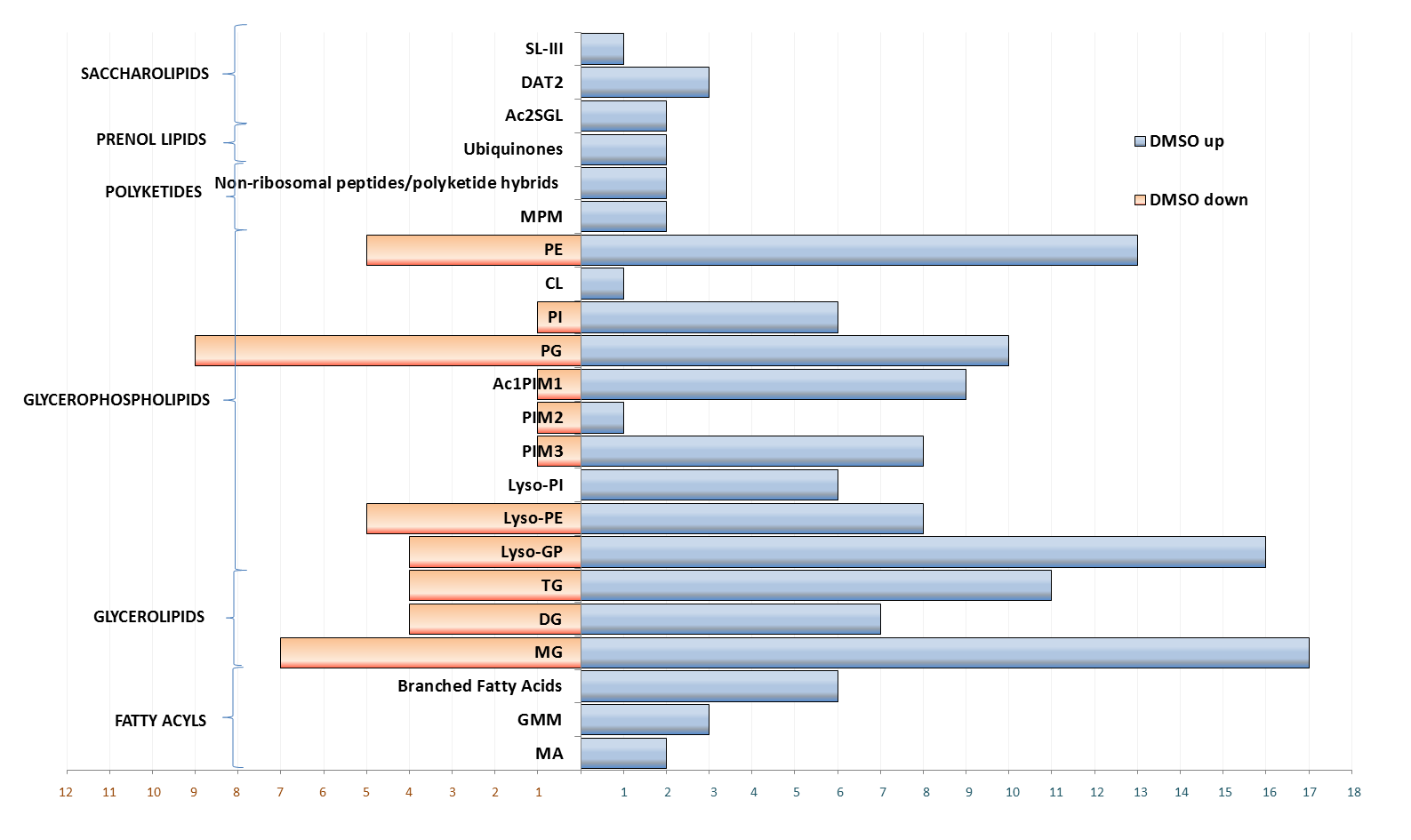


**Figure S1.** The number of different m/z upregulated or downregulated under the influence of DMSO (versus untreated) assigned to a given lipid class. Blue and red bars represent upregulated and downregulated features, respectively. MA–Mycolic acids; DIMA–Phthiocerol dimycocerosates; GMM–Glucose monomycolates; MBFA–Methyl branched fatty acids; MG–Monoacylglycerols; DG–Diacylglycerols; TG–Triacylglycerols; Lyso-GP–Monoacylglycerophosphoglycerols; Lyso-PE–Monoacylglycerolphosphoethanolamines; Lyso-PIM1–Monoacylglycerophosphoinositolmonomannosides; Lyso-PI–Monoacylglycerophosphoinositols; PIM4–Diacylglycerophosphoinositoltetramannosides; PIM3–Diacylglycerophosphoinositoltrimannosides; PIM2–Diacylglycerophosphoinositoldimannosides; Ac1PIM1–Monoacylated diacylglycerophosphoinositolmonomannosides; Ac1PIM3–Monoacylated diacylglycerophosphoinositolmonotrimannosides; Ac2PIM2–Diacylated diacylglycerophosphoinositoldimannosides; PG–Diacylglycerophosphoglycerols; PI–Diacylglycerophosphoinositols; CL–Diacylglycerophosphoglycerophosphodiradylglycerols; PE–Diacylglycerolphosphoethanolamines; MPM–Mannosyl-b1-phosphomycoketides; Ac2SGL–Diacylated Sulfolipid; DAT2–2,3-di-O-acyltrehaloses; DAT1–2,3-di-O-acyltrehaloses; SL-III–Sulfolipid III


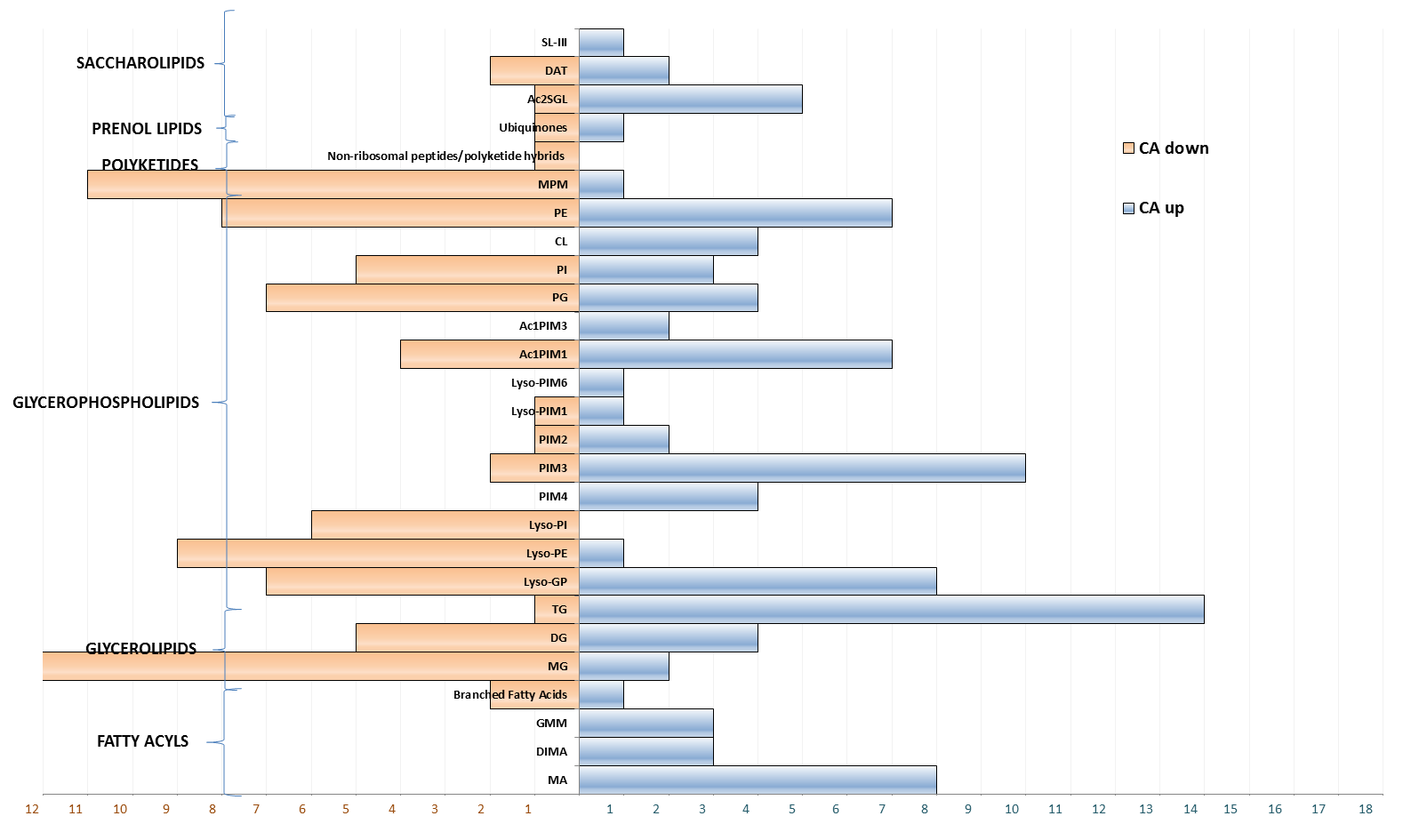


**Figure S2**. The number of different m/z upregulated or downregulated under the influence of CA (versus DMSO) assigned to a given lipid class. Blue and red bars represent upregulated and downregulated features, respectively. MA–Mycolic acids; DIMA–Phthiocerol dimycocerosates; GMM–Glucose monomycolates; MBFA–Methyl branched fatty acids; MG–Monoacylglycerols; DG–Diacylglycerols; TG–Triacylglycerols; Lyso-GP–Monoacylglycerophosphoglycerols; Lyso-PE–Monoacylglycerolphosphoethanolamines; Lyso-PIM1–Monoacylglycerophosphoinositolmonomannosides; Lyso-PI–Monoacylglycerophosphoinositols; PIM4–Diacylglycerophosphoinositoltetramannosides; PIM3–Diacylglycerophosphoinositoltrimannosides; PIM2–Diacylglycerophosphoinositoldimannosides; Ac1PIM1–Monoacylated diacylglycerophosphoinositolmonomannosides; Ac1PIM3–Monoacylated diacylglycerophosphoinositolmonotrimannosides; Ac2PIM2–Diacylated diacylglycerophosphoinositoldimannosides; PG–Diacylglycerophosphoglycerols; PI–Diacylglycerophosphoinositols; CL–Diacylglycerophosphoglycerophosphodiradylglycerols; PE–Diacylglycerolphosphoethanolamines; MPM–Mannosyl-b1-phosphomycoketides; Ac2SGL–Diacylated Sulfolipid; DAT2–2,3-di-O-acyltrehaloses; DAT1–2,3-di-O-acyltrehaloses; SL-III–Sulfolipid III

**Table S1**. Predictive metabolite results under cinnamaldehyde treatment.

| **Annotated Metabolites** | **Disregulation** | **Fold Change** | ***p*-value** |
| --- | --- | --- | --- |
| α-D-glucosamine 6-phosphate | UP | 5.7 | 2.7e-5 |
| 5-amino-6-(5-phospho-D-ribosylamino)uracil | DOWN | 5.5 | 3.6e-4 |
| 7,8-dihydrofolate | UP | 42.4 | 2.9e-5 |
| 7,8-dihydropteroate | UP | 9.7 | 3.8e-4 |
| D-glucosamine 1-phosphate | UP | 5.7 | 2.7e-5 |
| L-histidine | DOWN | 3.2 | 1.6e-4 |
| L-tyrosine | DOWN | 3.2 | 1.6e-4 |
| mycothione | DOWN | 4.6 | 8.2e-4 |
| precorrin-3B | UP | 21.2 | 6.6e-5 |
| protoporphyrin IX | DOWN | 2.3 | 7.0e-4 |
| S-adenosyl-4-methylthio-2-oxobutanoate | DOWN | 4.2 | 5.1e-10 |
| 5-amino-6-(D-ribitylamino)uracil | DOWN | 9.1 | 3.2e-8 |
| 2,5-diamino-6-(5-phospho-D-ribosylamino)pyrimidin-4(3H)-one | DOWN | 20.0 | 3.3e-6 |
| 5,10-methylene-tetrahydromethanopterin | UP | 162.3 | 8.8e-5 |
| 8-amino-7-oxononanoate | DOWN | 12.8 | 9.0e-5 |
| hydroxymethylbilane | UP | 55.0 | 9.1e-5 |
| uroporphyrinogen-III | UP | 55.0 | 9.1e-5 |

**Table S2**. Dysregulation in particular classes of detected lipids under the influence of cinnamon essential oil/cinnamaldehyde and DMSO. The numbers in brackets represent number of different m/z assigned to a given lipid class which were upregulated or downregulated.

| **Detected Compounds** | **Cinnamaldehyde**  **(up/down)** | **DMSO**  **(up/down)** |
| --- | --- | --- |
| **FATTY ACYLS** |  |  |
| Mycolic Acids (MA) | 6 (6/0) | 2 (2/0) |
| Phthiocerol Dimycocerosates (DIMA) | 4 (4/0) | 0 (0/0) |
| Glucose Monomycolates (GMM) | 2 (2/0) | 3 (3/0) |
| Branched Fatty Acids | 8 (4/4) | 6 (6/0) |
| **GLYCEROLIPIDS** |  |  |
| Monoacylglycerols (MG) | 15 (4/11) | 24 (17/7) |
| Diacylglycerols (DG) | 10 (3/7) | 11 (7/4) |
| Triacylglycerols (TG) | 23 (17/6) | 15 (11/4) |
| **GLYCEROPHOSPHOLIPIDS** |  |  |
| Monoacylglycerophosphoglycerols (Lyso-GP) | 17 (13/4) | 20 (15/5) |
| Monoacylglycerolphosphoethanolamines (Lyso-PE) | 9 (7/2) | 12 (7/5) |
| Monoacylglycerophosphoinositolmonomannosides (Lyso-PIM1) | 1 (1/0) | 0 (0/0) |
| Monoacylglycerophosphoinositolhexamannosides (Lyso-PIM6) | 1 (1/0) | 0 (0/0) |
| Monoacylglycerophosphoinositols (Lyso-PI) | 4 (2/2) | 0 (6/0) |
| Diacylglycerophosphoinositoltetramannosides (PIM4) | 4 (4/0) | 0 (0/0) |
| Diacylglycerophosphoinositoltrimannosides (PIM3) | 12 (12/0) | 9 (8/1) |
| Diacylglycerophosphoinositoldimannosides (PIM2) | 2 (2/0) | 3 (2/1) |
| Monoacylated diacylglycerophosphoinositolmonomannosides (Ac1PIM1) | 11 (11/0) | 11 (9/2) |
| Monoacylated diacylglycerophosphoinositoltrimannosides (Ac1PIM3) | 2 (2/0) | 0 (0/0) |
| Diacylated diacylglycerophosphoinositoldimannosides (Ac2PIM2) | 1 (1/0) | 0 (0/0) |
| Diacylglycerophosphoglycerols (PG) | 15 (10/5) | 18 (10/8) |
| Diacylglycerophosphoinositols (PI) | 7 (7/0) | 8 (7/1) |
| Diacylglycerophosphoglycerophosphodiradylglycerols (CL) | 4 (4/0) | 2 (2/0) |
| Diacylglycerolphosphoethanolamines (PE) | 21 (16/5) | 19 (13/6) |
| **POLYKETIDES** |  |  |
| Mannosyl-b1-phosphomycoketides (MPM) | 4 (3/1) | 4 (3/1) |
| Non-ribosomal peptides/polyketide hybrids | 1 (1/0) | 2 (2/0) |
| **PRENOL LIPIDS** |  |  |
| Ubiquinones [PR0201] | 2 (2/0) | 2 (2/0) |
| **SACCHAROLIPIDS** |  |  |
| Diacylated Sulfolipid (Ac2SGL) | 6 (6/0) | 2 (2/0) |
| 2,3-di-O-acyltrehaloses (DAT1) | 2 (0/2) | 1 (1/0) |
| 2,3-di-O-acyltrehaloses (DAT2) | 3 (3/0) | 3 (3/0) |
| Sulfolipid III (SLIII) | 0 (0/0) | 1 (1/0) |

**Table S3**. Lipid molecules with fold change > 10, altered during treatment with cinnamaldehyde and DMSO.

| **Metabolites with fold change >10** | **Formula** | **Fold Change** | ***p*-value** | **Stress Factor** |
| --- | --- | --- | --- | --- |
| **FATTY ACYLS** |  |  |  |  |
| Alpha Mycolic Acid (Alpha-MA) C91 | C91H178O3 | 19.81173882 up | 0.00068343 | CA |
| Alpha Mycolic Acid (Alpha-MA) C93 | C93H182O3 | 17.64299213 up | 0.001925244 | DMSO |
| **GLYCEROLIPIDS** |  |  |  |  |
| Triacylglycerol (TG) R1CO2H+R2CO2H+R3CO2H=72:0 (Glycerol tritetracosanoate) | C75H146O6 | 41.88481675 up | 0.000385487 | CA |
| Monoacylglycerol (MG) RCO2H=16:0 | C19H38O4 | 18.8011969 down | 0.028965937 | DMSO |
| **GLYCEROPHOSPHOLIPIDS** |  |  |  |  |
| Monoacylglycerophosphoglycerol (Lyso-GP) RCO2H=20:0 | C26H53O9P1 | 13.80827021 up | 6.35259E-05 | CA |
| Monoacylglycerophosphoglycerol (Lyso-GP) RCO2H=19:1 | C25H49O9P1 | 31.36688616 up | 2.49606E-05 | DMSO |
| Monoacylglycerophosphoglycerol (Lyso-GP) RCO2H=19:0 | C25H51O9P1 | 18.07826451 down | 0.025887803 | DMSO |
| Monoacylglycerophosphoglycerol (Lyso-GP) RCO2H=16:0 | C22H45O9P1 | 18.19394504 down | 0.028098256 | DMSO |
| Diacylglycerophosphoglycerol (PG)  R1CO2H+R2CO2H=29:1 | C35H67O10P1 | 72.66568606 down | 0.028175417 | DMSO |
| Monoacylated diacylglycerophosphoinositolmonomannoside (Ac1PIM1) R1CO2H+R2CO2H+R3CO2H=46:2 | C61H111O19P1 | 13.03744466 up | 0.000495444 | CA |
| Monoacylated diacylglycerophosphoinositoltrimannoside (Ac1PIM3) R1CO2H+R2CO2H+R3CO2H=55:4,R4=H | C82H145O29P1 | 11.30176179 up | 0.000751685 | CA |
| Monoacylglycerolphosphoethanolamine Lyso-PE R1CO2H=17:0 | C22H46N1O7P1 | 10.38054208 down | 0.036925482 | DMSO |
| Diacylglycerolphosphoethanolamine (PE) R1CO2H+R2CO2H=34:2 | C39H74N1O8P1 | 44.10851411 up | 0.007114591 | CA |
| **SACCAROLIPIDS** |  |  |  |  |
| Diacylated Sulfolipid (Ac2SGL) C60 | C60H114O17S1 | 23.24011815 up | 0.000154852 | CA |
| Diacylated Sulfolipid (Ac2SGL) C60 | C60H114O17S1 | 31.13575982 up | 9.4845E-05 | DMSO |

CA–cinnamaldehyde, DMSO–dimethyl sulfoxide.
